# Estimation of trophic niches in myrmecophagous spider predators

**DOI:** 10.1038/s41598-020-65623-8

**Published:** 2020-05-26

**Authors:** Lenka Petráková Dušátková, Stano Pekár, Ondřej Michálek, Eva Líznarová, William O. C. Symondson

**Affiliations:** 1 Department of Botany and Zoology, Faculty of Science, Masaryk University, Kotlářská 2, 61137 Brno, Czech Republic; 2 Cardiff School of Biosciences, Cardiff University, Sir Martin Evans Building, Museum Avenue, CF10 3AX Cardiff, UK

**Keywords:** Behavioural ecology, Ecology, Molecular ecology

## Abstract

Among spiders, taxonomically the most diversified group of terrestrial predators, only a few species are stenophagous and feed on ants. The levels of stenophagy and ant-specialisation vary among such species. To investigate whether stenophagy is only a result of a local specialisation both fundamental and realised trophic niches need to be estimated. Here we investigated trophic niches in three closely-related spider species from the family Gnaphosidae (*Callilepis nocturna*, *C. schuszteri*, *Nomisia exornata*) with different levels of myrmecophagy. Acceptance experiments were used to estimate fundamental trophic niches and molecular methods to estimate realised trophic niches. For the latter two PCR primer sets were used as these can affect the niche breadth estimates. The general invertebrate ZBJ primers were not appropriate for detecting ant DNA as they revealed very few prey types, therefore ant-specific primers were used. The cut-off threshold for erroneous MOTUs was identified as 0.005% of the total number of valid sequences, at individual predator level it was 0.05%. The fundamental trophic niche of *Callilepis* species included mainly ants, while that of *N. exornata* included many different prey types. The realised trophic niche in *Callilepis* species was similar to its fundamental niche but in *N. exornata* the fundamental niche was wider than realised niche. The results show that *Callilepis* species are ant-eating (specialised) stenophagous predators, catching mainly Formicinae ants, while *N. exornata* is an ant-eating euryphagous predator catching mainly Myrmicinae ants.

## Introduction

To understand trophic interactions in ecological, evolutionary or conservation studies a reliable estimate of the trophic niche is essential. For example, specialised exploitation of a limited type of resources (or stenophagy) can be either due to local (ecological) or global specialisation^1,2^. This can be resolved by comparing the fundamental and realised niches. While the former trophic niche is wide as it includes all prey types that the predator is capable of exploiting, the latter is obviously narrower as it is limited by prey availability. The fundamental niche can only be measured experimentally, whereas the realised niche is inferred from field data where prey exploitation is determined by the spatial and temporal co-occurrence of predator and prey, inter- and intraspecific competition among predators, mutualism, etc.^2^. Thus to understand breadth of realised trophic niche of a predator, data on the available prey, co-occurring predators and competitors as well as local conditions are necessary.

Spiders are the most diversified and important terrestrial arthropod predators^3^ but evidence of breadth of trophic niche is available only for less than 2% of species^4^. Among these the majority of data exist for euryphagous species, i.e. feeding on wide variety of prey^5^. Trophic niche of stenophagous species has been little investigated because of largely cryptic feeding (i.e. hidden in the litter), thus it is not known whether and to what extent they are specialised on a certain prey. The levels of specialisation vary and are closely linked to behavioural, morphological, and/or physiological adaptations^6^ which can only be revealed by means of experimental approaches^7^. Alternatively, the level of specialisation can be deduced by comparing fundamental and realised trophic niches. Thanks to the slow metabolic rate of spiders compared to other invertebrates^8,9^, the natural diet (the realised trophic niche) might be reconstructed even several weeks after food intake using molecular methods^10^.

For species with cryptic feeding behaviour, gut content analysis based on prey DNA may be very efficient at determining the realised trophic niche of a species^11–13^. However, the results may be affected, for example, by the use of PCR primers, sequences filtering and clustering. In order to detect a wide range of prey, a number of “universal” primers for prey DNA detection have been developed to date^14–21^. However, the primers differ in the efficacy with which they amplify the target groups^22^. Moreover, PCR and sequencing artefacts or tag jumping, which arise at different steps of the gut content analysis and the targeting of highly degraded prey DNA, can affect the quality of the results^23^.

Selection of the appropriate thresholds for MOTU (molecular operational taxonomic units) clustering can affect the estimates of the niche breadth as it can differ between markers^24^. Consequently, the number of species detected can be over- or underestimated^25^. Finally, removing singletons and also MOTUs represented by low number of reads could help to obtain more reliable results^26,27^ as rare MOTUs often represent erroneous sequences or chimeric reads^28,29^.

Available data show that the most common specialisation among stenophagous spiders is myrmecophagy (ant-eating^4^). Evidence for this type of specialisation is rare and of insufficient quality^4^. Thus, in this study, we aimed to estimate the fundamental and realised trophic niches of three closely related European spider species (Araneae: Gnaphosidae) for which available (anecdotal) data indicate various levels of stenophagy/specialisation on ants^30,31^. Two of the species (*Callilepis* spp.) have sympatric distribution in central Europe while the other (*Nomisia exornata*) occurs in the Mediterranean^30,31^. We performed laboratory experiments to identify breadth of their fundamental trophic niche, detected their prey DNA using two different primer pairs (general invertebrate and ant-specific) and estimated the breadth of realised trophic niches. Finally, we performed simulations to find out how different thresholds for excluding rare MOTUs can affect estimates of realised trophic niche breadths.

## Results

### Fundamental trophic niche

**Figure 1:**
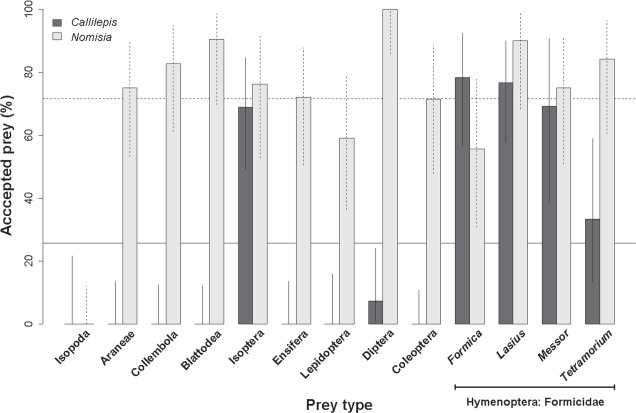
Comparison of the relative frequencies (percentage) with which 13 prey types were accepted by *Callilepis* spp. and *Nomisia exornata* in the laboratory. Solid horizontal line shows the overall mean of prey acceptance for *Callilepis* spp., dashed line for *N. exornata*. Vertical lines represent 95% confidence intervals.

In the laboratory, *Callilepis* spp. accepted three out of the ten offered prey orders (13 prey types, Table 1) but at significantly different frequencies (GEE, χ^2^_12_ = 41084, P < 0.0001). The average prey acceptance of all 13 prey types was 26%. *Callilepis* spp. did not accept woodlice, spiders, springtails, cockroaches, crickets, caterpillars, or beetles. It accepted fruit flies (Binomial test, P = 0.03) at a significantly lower frequency than average, and termites and ants (Binomial tests, P < 0.01) at a significantly higher frequency. The exception was *Tetramorium* ants, which were accepted at the average (no choice or random) frequency (Binomial test, P = 0.43, Fig. 1).

**Table 1:** List of prey types used in acceptance experiments, their body size (*prosoma size otherwise total body size) and the number of offered prey (N).

Order/Family	Species	Prey size (mm)	N	
			*Callilepis*	*Nomisia*
Isopoda	*Porcellio scaber* Latreille, 1804;	5.00 ± 1.08	17	28
	*Armadillidium vulgare* Latreille, 1804			
Araneae	*Clubiona* sp.; Araneidae juveniles	1.65 ± 0.30*	25	24
Collembola	*Sinella curviseta* Brook, 1882	1.50 ± 0.00	27	23
Blattodea	*Symploce pallens* (Stephens, 1835)	3.20 ± 1.01	28	21
Isoptera	*Reticulitermes santonensis* Feytaud, 1924;	3.87 ± 0.46	29	21
	*Reticulitermes flavipes* (Kollar, 1837)			
Ensifera	*Acheta domestica* (Linnaeus 1758)	3.10 ± 0.25	25	25
Lepidoptera	*Ephestia kuehniella* Zeller, 1879, caterpillars	5.00 ± 1.42	21	22
Hymenoptera: Formicidae	*Lasius niger* (Linnaeus, 1758);	4.06 ± 0.49	30	20
	*Lasius alienus* Förster, 1850			
	*Formica* sp.	5.52 ± 0.82	23	18
	*Tetramorium* sp.	2.79 ± 0.34	18	19
	*Messor* sp.	5.54 ± 1.16	13	20
Diptera	*Drosophila melanogaster* Meigen, 1830;	2.00 ± 0.32	27	24
	*Drosophila hydei* Sturtevant, 1921			
Coleoptera	*Callosobruchus maculatus (*Fabricius, 1775)	3.00 ± 0.27	32	21

*Nomisia exornata* accepted nine of the ten offered prey orders at significantly different frequencies (GEE, χ^2^_12_ = 98597, P < 0.0001). The average prey acceptance of all prey types was 72%. It did not accept woodlice. Fruit flies were accepted at a significantly higher frequency than average (Binomial test, P < 0.001). There was also a trend showing a higher acceptance for cockroaches and *Lasius* ants (Binomial tests, P < 0.1). Other offered prey types were accepted at average frequencies (Fig. 1).

Levins’ index of fundamental niche breadth for *N. exornata* was high (*B*_*A*_ = 0.87), while for *Callilepis* spp. it was low (*B*_*A*_ = 0.13). Thus *Callilepis* spp. is stenophagous, capturing predominantly ants, whereas *N. exornata* is euryphagous but also catching ants.

### Realised trophic niche

The ZBJ primers amplified all potential ant prey species (N = 11) except *Tetramorium* sp. but failed to amplify *Acheta domestica* and *Symploce pallens* in the lab testing. However, the use of ZBJ primers did not reveal the ant prey inside the predators’ guts except for one individual of *C. schuszteri*. Here *Camponotus vagus* ant was detected using ZBJ primers in spite of the fact that in almost all other predators the ants were detected using the ant primers (see below). Prey other than ants detected in the spider guts using general invertebrate primers was very scarce, represented by Diptera, Lepidoptera and Collembola (assigned to a family or order level only).

All potential ant prey species (N = 11) were successfully amplified using the ant specific primer when testing it in the lab. We detected 13 ant genera and one representative of the family Crabronidae (Hymenoptera) in the guts of the studied spiders.

Prey DNA was successfully amplified from the guts of 22 out of 24 *C. nocturna* (Linnaeus, 1758) individuals (Table S1), 40 out of 48 *C. schuszteri* (Herman, 1879) individuals (Table S2), and in all (N = 72) *N. exornata* (C. L. Koch, 1839) individuals (Table S3). In total we obtained 3,454,734 reads from the *Callilepis* sequencing run and 3,322,943 reads from *N. exornata* sequencing. From those numbers 30.9% and 36% of the total sequencing output were informative (=sequences of correct length, assigned to either predator or prey) sequences in *Callilepis* spp. and *N. exornata*, respectively (Table 2). Despite the use of blocking oligo preventing predators DNA amplification, most of the sequences obtained using the ZBJ primers belonged to the predators: only 0.6% of those reads in *Callilepis* spp. and 13.2% in *N. exornata* belonged to a prey.

**Table 2:** Number of sequences and MOTUs in different steps of the analysis.

Predator	Seq. output	# prey sequences		# predator seq.	# prey MOTUs		# predator MOTUs	# prey MOTUs (0.005% cut-off applied)	# prey genera (0.5% cut-off/no cut-off applied)	
		Form	ZBJ	ZBJ	Form	ZBJ	ZBJ	Form	Form	ZBJ
*Callilepis*	3,454,734	1,068,419	668	110,554	75	5	3	28	9/10	1
*C. nocturna*		421,287	2	83,589	44	1	1	19	7/10	—
*C. schuzsteri*		647,132	666	26,965	62	4	2	19	6/10	1
*N. exornata*	3,322,943	405,634	32,625	57,935	50	10	5	21	8/10	—

As both *Callilepis* species have been sequenced together in one sequencing run we present also the sum of both. (Form = ant specific primers, ZBJ = general invertebrate primers, Seq. output = total no. of reads in each sequencing run).

**Figure 2:**
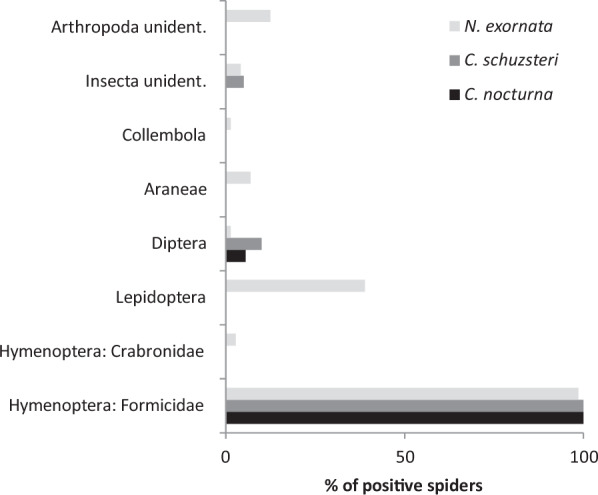
Comparison of proportions of field-collected individuals of two *Callilepis* species and *N. exornata* positive for DNA of different prey types.

**Figure 3:**
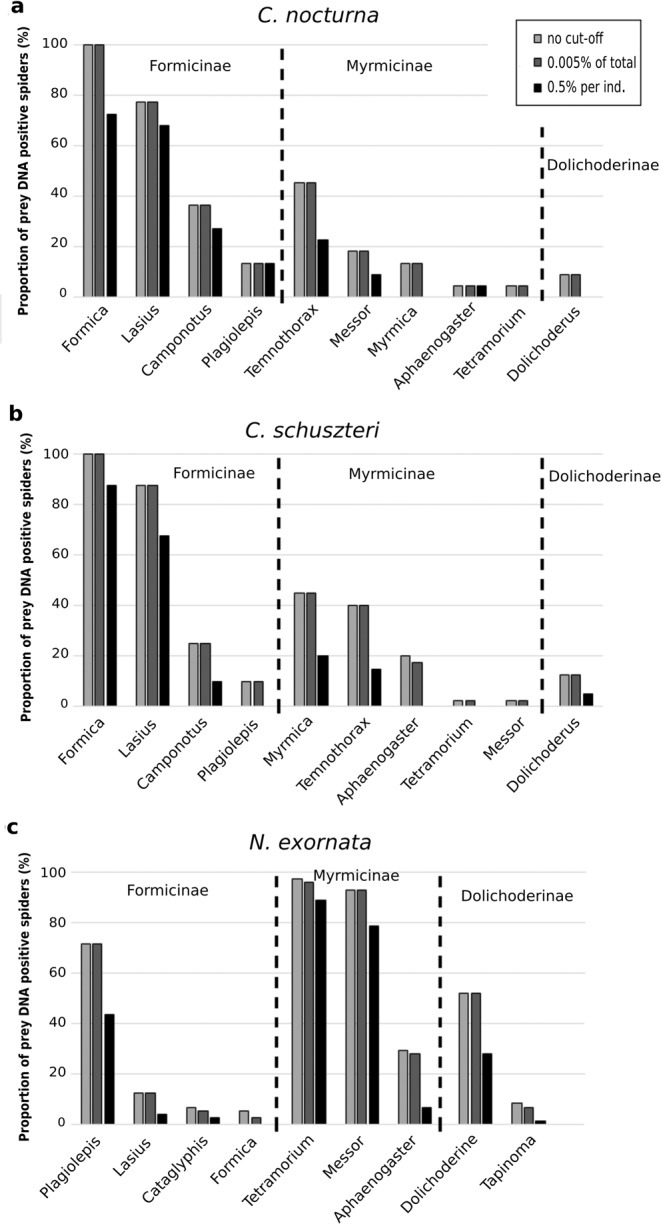
Percentage of ant genera found in *Callilepis* spp. and *N. exornata* guts – a comparison among different data treatment: no sequence excluded (grey), data after excluding those MOTUs which contained less sequences than 0.005% of the total valid reads (dark grey), and data after excluding those prey types which was represented by less sequences than 0.5% of the valid sequences obtained from an individual spider (black).

Ants formed the majority of prey detected in all three spider species (Fig. 2). In *N. exornata*, we detected nine ant genera and an unidentified crabronid wasp, ten ant genera were found in both *C. nocturna* and *C. schuszteri*. After applying a cut-off for excluding prey represented by low sequence number (per individual), eight, seven and six ant genera remained in *N. exornata*, *C. nocturna*, and *C. schuszteri*, respectively (Fig. 3).

Formicinae ants were predominant among ants consumed by *C. nocturna* and *C. schuszteri* (100% of all ant DNA positive spiders), while Myrmicinae ants were predominant in *N. exornata* (98.6% of all ant DNA positive spiders, Fig. 3).

In four *C. schuszteri* individuals and one *C. nocturna* individual, the non-ant prey was only represented by Diptera (most likely Heleomyzidae). In two *C. schuszteri* individuals unidentified insect was detected. In *N. exornata* we identified Lepidoptera in 28 individuals, Diptera (Cecidomyiidae) in one individual and Collembola in one individual. In five individuals we found unidentified Araneae. In twelve cases the prey was assigned as unidentified insect or unidentified arthropods as the sequences were identical to several orders.

The Shannon and Levins’ indices based on the ant primers was equal to zero in all three species as only Hymenoptera could be detected using those primers.

### Cut-off thresholds

**Figure 4:**
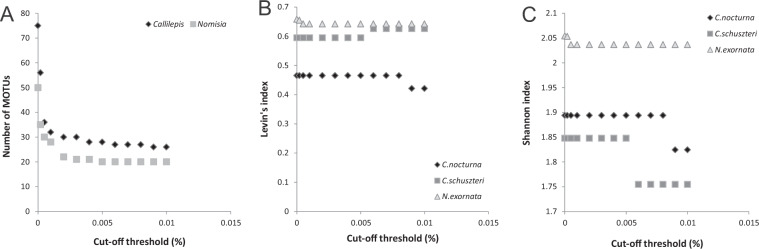
Relationship between the number of MOTUs (**A**), Levin’s (**B**) and Shannon (**C**) diversity indices of prey detected in the predators and cut-off thresholds applied to the whole dataset. Indices were estimated at the prey genus level. The MOTUs containing fewer sequences than was a threshold value (percentage of the total valid sequence numbers) were excluded.

The Levin’s and Shannon indices, estimated at the prey genus level, were constant between 0.001–0.005% cut-off thresholds in all three studied species while number of MOTUs was decreasing (Fig. 4). The steepest decrease in MOTUs number was found between the 0–0.0005% cut-off, then the number of MOTUs decreased less markedly. Taking into account all these features, we suggest the threshold of 0.005% (from the total number of valid reads) as optimal for excluding rare (erroneous) MOTUs.

**Figure 5:**
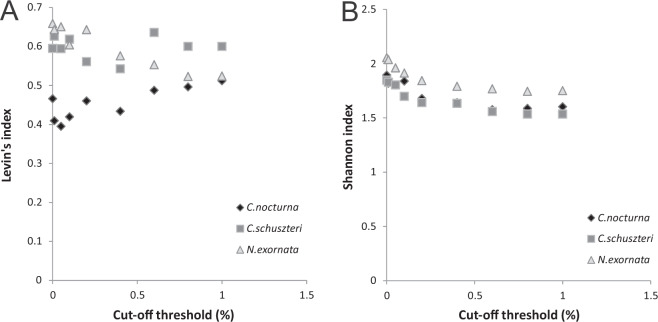
Relationship between the Levin’s (**A**) and Shannon (**B**) indices of prey detected in the predators and cut-off threshold applied to each individual spider data. Indices were estimated at the prey genus level. The MOTUs containing fewer sequences than was a threshold value (percentage of the number of valid sequences found in each predator) were excluded.

When applying cut-off thresholds for each individual separately, the Shannon index decreased across the whole range of the selected thresholds in *C. schuszteri and N. exornata*. Using the Levin’s index we did not find any clear pattern (Fig. 5). In all three species the indices did not change in cut-offs higher than 0.5%, therefore we applied the 0.5% threshold. However, this cut-off value markedly reduced the index values and the number of prey genera detected (compared to non-filtered data). On the other hand, the prey which is represented by number of sequences above this threshold could be reliably identified. Most of all unidentified ant (to genus) prey disappeared after applying the cut-off.

The index values were almost identical when no cut-off was applied and MOTUs containing less than 0.005% of the total number of valid sequences were removed (Table 3).

**Table 3:** Shannon and Levin’s diversity indices calculated for ant prey at genus level detected in the spider predators in three different data treatments (no cut-off, MOTUs represented by less than 0.005% of the total valid sequences excluded, MOTUs represented by less than 0.5% of the sequences obtained from each individual predator excluded).

Treatment	Levin’s index			Shannon index		
	*C. nocturna*	*C. schuzsteri*	*N. exornata*	*C. nocturna*	*C. schuzsteri*	*N. exornata*
no cut-off	0.4502	0.4747	0.4681	1.8613	1.8709	1.8169
0.005% cut-off	0.4019	0.4686	0.4634	1.8088	1.8643	1.8122
0.5% cut-off	0.5234	0.4484	0.3618	1.6116	1.3855	1.5472

## Discussion

Our results from the prey DNA analysis show that both *N. exornata* and *Callilepis* spp. spiders hunt mostly ants in the field. Results from the acceptance experiments suggested that *Callilepis* spp. spiders rarely accepted other prey than ants; it is thus a stenophagous predator, whereas *Nomisia* frequently accepted other prey types, it is thus a euryphagous predator (*sensu*^6^).

Prey other than ants was found mainly in *N. exornata* spiders. However, the prey detected in *N. exornata* guts did not correspond to the wide range of prey accepted by the spiders in the acceptance experiments. The disagreement could be caused by the efficacy of the general invertebrate primers as well as prey availability. We detected only Diptera, Lepidoptera and Collembola in their guts, while in the acceptance experiments *N. exornata* fed also on Blattodea and Ensifera with higher frequency than on Lepidoptera. We failed to amplify representatives of Blattodea and Ensifera when testing the primers in the lab and thus these prey types remained undetectable (Table S4). By contrast, prey other than ants was very scarce in *Callilepis* spp. guts, represented only by Diptera, and was detected in very low sequence numbers, suggesting this prey could rather represent a case of secondary predation^8,32,33^.

While both *Callilepis* species preyed mainly on Formicinae ants, *N. exornata* preyed mainly on Myrmicinae ants. Similarly, in the acceptance experiments, *N. exornata* accepted slightly more Myrmicinae than Formicinae ants, while in *Callilepis* it was the other way around. This suggests that *Callilepis* is rather specialised on Formicinae and *N. exornata* on Myrmicinae ants. It also reflects the different spider and ant species distribution patterns: the species richness of Myrmicinae ants is higher in southern (where *N. exornata* occurs) than in central Europe (where *Callilepis* spp. occur)^34^. In addition, *N. exornata* is nocturnal (Pekár, unpublished), while species of *Callilepis* are diurnal. Indeed, some Myrmicinae ants are more inclined to be nocturnal or dusk foragers, while some Formicinae ants are diurnal^35,36^.

The two *Callilepis* species turned out to have very similar realised trophic niches. But a closer look revealed that *Callilepis nocturna* captured slightly more *Camponotus* and *Messor* ants than *C. schuzsteri* which instead captured more *Myrmica* and *Aphaenogaster* ants. As the two species may occur together there is a room to avoid competition by exploiting different ant species.

In the acceptance experiments, *Callilepis* spp. accepted *Formica*, *Lasius*, and *Messor* with high frequency, while *Tetramorium* was a less frequently accepted. Similarly, in the field *Formica* and *Lasius* were the most common prey, while *Tetramorium* was rarely found in their guts. Isoptera were widely accepted in the lab but were not encountered in the field, Diptera were rarely accepted in the lab or field. In *N. exornata*, *Tetramorium* and *Messor* were frequently accepted in the lab and also in the field, while *Formica* was the least predated in both cases. While *N. exornata* accepted a wide range of prey offered in the lab, only a few Lepidoptera, Diptera, and Collembola were found in their guts besides Hymenoptera. To summarize, the fundamental and realised niches were similar in *Callilepis* spp. but different in *N. exornata*, where the realised niche was narrower than in the fundamental niche. This is a common phenomenon caused by local (ecological) stenophagy^37^.

The ZBJ primers^18^ have been used more extensively than any other general primers in diet studies^38–40^. Although these were originally developed for bat trophic interactions with invertebrate prey, they have also been used successfully with other predators^19,33,41,42^. It is known that these primers preferentially amplify certain groups, such as Diptera and Lepidoptera, and may lead to a strong bias in niche breadth estimates^20,43^. The primers might not reveal all target taxa due to preferential PCR amplification of some, which are either more abundant in the DNA mixture or a better fit to the primers as there are few mismatches in the primer region^21^. Indeed, the predators had fewer mismatches in the ZBJ primers than the tested ants. The primers were tested with good quality DNA but in spiders’ guts the prey DNA was highly degraded, in negligible amounts in comparison to the predator DNA. Although we used blocking oligos to prevent predators’ DNA amplification, the majority of the sequences found belonged to the predators. Even at high concentration of the C3 spacer blocking oligo not all predators DNA amplification is usually “blocked”^43^. Some prey types can be blocked when the prey DNA is similar to that of the predators in the target region, thus we avoided too high blocking oligo concentration. As two different amplicon lengths were sequenced in the same sequencing run, shorter fragments (obtained from ant primers) could be preferentially amplified during PCR. Ants were detected only in one *Callilepis* spp. individual using ZBJ primers, though the ant primers allowed ant DNA detection in almost all individuals. ZBJ primers^18^ turned out to be useless for ant DNA detection and, consequently, for diet studies in myrmecophagous animals. On the other hand, the primers worked well with other invertebrate prey, such as Diptera, Lepidoptera and spiders, as revealed in the study of araneophagous spiders^33^.

Total number of MOTUs detected as prey of *Callilepis* spp. and *N. exornata* was high. The number of MOTUs assigned to a genus was even higher than the number of species of that genus recorded in the ant checklists^44,45^. We assume that including all (non-filtered) data artificially increases prey diversity. Moreover, when no cut-off was applied we got substantially higher number of the MOTUs which could not be identified to genus level. The largest reduction in detected diversity was introduced by singleton removal^24^ and removal of MOTUs occurring only once in the dataset^25^. We recommend deletion not only of singletons but also the MOTUs with the lowest number of sequences as they probably also represent erroneous reads. To check whether the steepest decrease in MOTUs number depends on number of reads in MOTUs may help to identify which MOTUs could be excluded. In our study, approximately a half of all MOTUs disappeared after removing the MOTUs containing less than five sequences (corresponding to 0.0005% from the whole dataset). Absolute threshold of five seemed to be the most effective also based on analysis of the replicates^24^. In cut-offs higher than 0.002% the number of MOTUs was approaching a plateau and the estimation of trophic niche indices was not affected. In another study^21^ the MOTUs which had more than 0.003% of the total number of the valid sequences were considered reliable, otherwise they were excluded from the data. However, the authors provided no explanation why they selected these thresholds. We selected the threshold 0.005% (of the whole data) as optimal because with higher cut-off the diversity indices  decreased in *Callilepis* spp. The use of relative threshold for each individual predator also makes sense because the number of reads per individual predator can vary substantially. Different thresholds for different samples depending on the sequencing depth have been suggested^46^, e.g. haplotypes represented by less than 1% of the total number of reads in each specimen were removed^47^. However, using these relative thresholds could decrease prey diversity found in predators with very high number of prey sequences, and rare species might be neglected.

To eliminate errors it is also important to match the prey found in the guts to the data on occurrence of those species at the study site. Two genera, *Messor* and *Aphaenogaster*, which have not been reported from the study site^44^ were rarely found in *Callilepis* spp. guts. However, they are known from Austria from similar habitats and *Aphaenogaster* in particular might be overlooked due to its cryptic way of life. Although *Callilepis* spp. spiders have been observed feeding on *Camponotus vagus*^48^, we did not confirm that this ant species represents a dominant ant prey. As for *N. exornata* all ant genera found in the spiders guts are known from the sites of occcurence^45,49^.

To conclude, it is necessary to use ant-specific primers when investigating gut content of myrmecophagous predators by means of molecular methods as the ZBJ primers^18^ do not allow ant DNA detection in the gut. Alternatively, other general primers developed^50^ recently could be used. Furthermore, if the data on prey come from a large sample size it is advisable to use a cut-off threshold for excluding the MOTUs represented by low number of sequences when we want to obtain more reliable results (especially in case of Ion Torrent sequencing), although these results might not be complete – we might not reveal rare prey items. On the other hand, dealing with prey which occur in one or two predators and which was found in low sequence numbers may be risky as those prey types could represent contaminants or secondary prey. Rare prey (found in less than 5% of the studied predators) obviously do not represent important food source for the predators which would limit the predator’s population growth, so missing rare prey items has negligible impact on the species ecology^19^. In any case, a combination of different approaches may help to obtain more reliable results.

## Methods

### Sample collection

Two species of *Callilepis* were hand-collected on the forest edge at two sites. *Callilepis nocturna* and a few *C. schuszteri* spiders at various growth stages were collected in the valley of the Gröβer Dürrenbach river, between Villach and Klagenfurt, Austria in June 2015. *Callilepis schuszteri* individuals were also collected in Senorady, Czech Republic in June 2016. *Nomisia exornata* spiders at various stages were collected near Serpa, southern Portugal in October 2015 and 2017. Although the adults have been identified as *N. exornata*, the molecular data suggest that the studied individuals could represent two cryptic species. The potential prey (sympatrically occurring ants) were collected together with the spiders: *Lasius flavus* (Fabricius, 1782), *Lasius platythorax* Seifert, 1991, *Camponotus vagus* (Scopoli, 1763), *Tetramorium* sp., *Myrmica* sp., and *Temnothorax* sp. were collected at the same site as *Callilepis* spp. spiders. *Tetramorium semilaeve*, *Camponotus aethiops* (Latreille, 1798), *Cataplyphis hispanica* (Emery, 1906), *Messor barbarus* (Linnaeus, 1767), and *Aphaenogaster senilis* Mayr, 1853 were collected as the potential prey of *N. exornata* spiders. Collected ants and spiders for molecular gut content analysis were, immediately after hand-collection, stored in 100% ethanol. Spiders used in laboratory experiments were kept in plastic vials containing moisturized gypsum and placed in a chamber at a constant temperature (22 ± 1 °C) and under a LD regime (16:8). Spiders were fed at least once a week with an ant or were allowed to consume the prey accepted in laboratory trials. Experiments were performed from July 2015 to October 2017.

### Fundamental trophic niche

To investigate the fundamental trophic niche, acceptance trials were performed with *Callilepis* spp. (N = 42) and *N. exornata* (N = 47). A few juveniles of the two *Callilepis* species were used which were not identified to species level thus these data were pooled for *Callilepis* spp. Spiders were starved for one week before being used in trials. Individuals were placed singly in Petri dishes (diameter 5 cm). The trials began after at least 1 h of acclimation. Thirteen prey types (four ant species representing different genera and nine types of other arthropods, Table 1) were offered to each spider in a randomised order. Each prey type was offered to individual spiders only once. If the prey was not attacked within one hour, it was replaced with a different prey type. The trial ended when the spider killed and consumed the prey. If a spider did not accept any prey type, it was considered unmotivated to eat (i.e. satiated or preparing to moult) and such data were rejected. Trials were performed approximately at one week intervals.

The overall difference in acceptance rates for 13 prey types was analysed for both *Callilepis* spp. and *N. exornata* using Generalised Estimating Equations (GEE) from the geepack package^51^. GEE is an extension of the Generalised linear model (GLM) for correlated data. It was used because there were repeated measurements on each individual spider^52^. An AR1 correlation matrix was used to account for these temporal replications. Subsequently, the acceptance (i.e. the relative frequencies of acceptance) of each prey type was compared to the average prey acceptance by *Callilepis* spp. and *N. exornata* using a binomial test. The standardized Levins’ index (*B*_*A*_) of niche breadth^53^ was used to calculate the fundamental trophic niche breadth of *Callilepis* spp. and *N. exornata*. The index can reach values between 0 and 1. Ten prey types instead of 13 were used to calculate the index (all ant species were pooled and considered as a single category) so that the prey categorisation was homogeneous.

### Realised trophic niche

The realised trophic niche in *C. nocturna* (24 individuals), *C. schuszteri* (48 individuals), and *N. exornata* (72 individuals) spiders was investigated using gut content meta-barcoding. To detect the prey inside predators’ guts we used two types of PCR primers. General invertebrate primers^18^ (ZBJArtF1c: 5′-AGATATTGGAACWTTATATTTTATTTTTGG-3′ and ZBJArtR2c: 5′-WACTAATCAATTWCCAAATCCTCC) were used to amplify a wide range of taxa, while ant primers^54^ (ZodFormF: 5′-TTTATTAATRAWGGAGYAGGAACAGG and ZodFormR: 5′-CCTAARATTGAAGATATWCCTGCAAT) were used specifically to amplify ants only. The ZBJ fragment was positioned in the first third of the COI region (base positions 17-227 when COI amplified with Folmer *et al*.^55^ primers), the ZodForm fragment was positioned approximately in the half of the COI region (base positions 327- 457). Both primer pairs were tested with all field-captured ants and predator species to ensure that the primers enabled the amplification of all potential prey species, and to identify optimal PCR conditions (see below).

In all studied spider species and their potential ant prey, we amplified part of the cytochrome c oxidase gene using LCO1490 and HCO2198 primers^55^ and sequenced it on an ABI Prism 3130 Genetic Analyzer (Applied Biosystems). Sequences are deposited in GenBank. On the basis of COI gene sequences we designed blocking oligos for *Callilepis* spp. and for *N. exornata* spiders with C3 spacer modification at the 3′ end to minimize the predator DNA amplification (BLKCall: 5′-CAAATCCTCCAATCAAAATAG - C3 spacer) when using “universal” PCR primers and to increase the probability of prey DNA amplification. The blocking oligo originally designed for *Callilepis* spp. worked well also with *N. exornata* as they are phylogenetically closely related. However, optimal PCR conditions for the blocking oligo inhibting the predators DNA amplification and optimal PCR conditions for amplification of a range of potential prey differed (the blocking oligo had slightly higher annealing temperature than was the optimum for the universal primer). To allow the amplification of all potential prey we set up “suboptimal” PCR conditions for the predator DNA blocking, resulting in low efficacy of the blocking oligo (Table S4).

DNA from the guts of *Callilepis* spp. and *N. exornata* spiders was extracted using the DNeasy Blood & Tissue Kit (Qiagen, Hilden, Germany). Spider opisthosomas were crushed and incubated overnight with Proteinase K at 56 °C. PCR reactions with the ant specific primers were performed using Multiplex PCR kit (Qiagen) under the following conditions: initial denaturation at 95 °C for 15 min; 42 cycles of 94 °C for 30 s, annealing temperature (48 °C when using universal primers, 50 °C when using ant primers) for 90 s, 72 °C for 90 s; and a final extension at 72 °C for 10 minutes. The reaction mixture consisted of Qiagen Multiplex PCR Master Mix (10.6 μl), Q-Solution (1.8 μl), RNase-free water (2.8 μl), 10 μM forward and reverse primers (0.5 μl each), and DNA (7 μl). When using the universal primers, 1.2 μl of 100 μM blocking oligo was added to the reaction mixture. The PCR primers were tagged with MID identifiers (10-bases-long barcoding sequences) and each sample was PCR amplified using primers with a unique combination of MIDs on the forward and on the reverse primer. This allowed us to assign all DNA reads to each individual predator. PCR products were detected by electrophoresis in 2% GoodView-stained agarose gels and purified using QIAquick PCR Purification Kit (Qiagen)^33^. Enrichment (emPCR) and sequencing on the Ion Torrent platform with an Ion 318 chip and 400-base read length chemistry was provided by the Centre de Recerca en Agrigenòmica (Bellaterra, Spain).

The sequencing output was processed using the Galaxy platform (https://usegalaxy.org/), BioEdit 7.2.5^56^, fastx-toolkit and the EMBOSS packages^57^. The datasets were filtered for quality with Phred Q Score threshold 20. Then, reads were split according to their unique MID combinations, resulting in files corresponding to predator individuals. MIDs were then removed and too short and too long reads were excluded. The reads were collapsed and rare haplotypes (containing < 2 identical reads) were removed. Sequences were translated to amino acids according to the invertebrate mitochondrial genetic code and those containing stop codons were excluded as they represented nonsensical sequences. Sequences with insertions and deletions causing reading frame shifts were also removed. The remaining sequences were clustered into MOTUs using swarm^58^ with a 4-bp cut-off (=3% of the sequence divergence) in the case of specific ant primers, and with a 5-bp cut-off (=2.3%) for the ZBJ primers.

Each MOTU was compared to the GenBank database (https://blast.ncbi.nlm.nih.gov/Blast.cgi) using megablast, to the BOLD database (https://www.boldsystems.org/), and to the ant sequence library obtained from the potential prey. We identified the prey based on the closest match to reference sequences available in the BOLD and NCBI databases. When a sequence was more than 98% similar to one species, the prey was identified to the given species. When the sequence was more than 93% similar to one genus, it was assigned to the genus. Similarity to several different genera allowed the prey to be classified only to family level (e.g. Formicidae). In most cases we failed to assign the prey to species level thus the prey was assigned to a genus level only. The relatively low similarity level was probably related to lower sequences quality related to the sequencing technology used^59^, lack of records in the databases and intraspecific variability in the target taxa. In *Callilepis* spp. spiders, bacterial (Proteobacteria) and fungal (Ascomycota) DNA sequences were occasionally amplified using the ant primers, but they were excluded from the subsequent analysis as they do not represent actual prey.

The remaining sequences were then converted to binary (incidence) data, representing the number of spider individuals positive for DNA of particular prey types (at genus level for ants and at order level for other prey). Subsequently, the proportions of prey DNA positive spider individuals were estimated from the binary data. To estimate the breadth of the realised trophic niches we used both Levin’s and Shannon indices^60,61^.

### Cut-off thresholds

As another bias might arise during sample processing (erroneous sequences, incorrectly assigned prey to single predators because of cross-contamination, tag jumping, or secondary prey amplification), we estimated the trophic niche width using number of prey types (at genus level) by means of Shannon and Levin’s indices, while rejecting prey MOTUs which contained fewer sequences than was a defined cut-off threshold value. We determined relative thresholds for the total number of valid sequences obtained from each sequencing run (*Callilepis* spp. and *N. exornata*; 0, 0.0002%, 0.0005%, 0.001%, 0.002%, 0.003%, 0.004%, 0.005%, 0.006%, 0.007%, 0.008%, 0.009%, and 0.01% of the valid sequences) and relative thresholds for each individual predator (0, 0.01%, 0.05%, 0.1%, 0.2%, 0.4%, 0.6%, 0.8%, 1% of the valid sequences assigned to each predator individual), as the number of reads per individual varies. This comparison was done in twenty spiders of each species – we selected individuals in which more than five MOTUs were identified.

Based on the differences in Shannon and Levin’s index values and number of MOTUs for each cut-off threshold the optimal thresholds were selected for further analysis where all sequenced predator individuals were included. We then compared the indices and relative frequency of prey found in the predators guts for three different treatments: (1) no MOTUs excluded, (2) excluding MOTUs which contained less sequences than 0.005% of the total valid reads (potentially erroneous sequences removed), and (3) data after excluding those prey types which were represented by fewer sequences than 0.5% of the valid sequences obtained from an individual spider (prey which could be wrongly assigned to the predator individual due to potential tag jumps or secondary predation).

## Supplementary information

Supplementary Information.

## Data Availability

DNA sequences of COI gene used for primer evaluation are available in Genbank (accession numbers MG840340-MG840351, KX954280, KX954282, KX954293).
