# Supplementary material for: Estimation of trophic niches in myrmecophagous spider predators
Source: Sci Rep. 2020 May 26;10:8683. doi: 10.1038/s41598-020-65623-8 (PMC7250852; doi:10.1038/s41598-020-65623-8)
Supplement: Supplementary file 1 — Supplementary Information. [file 41598_2020_65623_MOESM1_ESM.docx]

Estimation of trophic niches in myrmecophagous spider predators

Lenka Petráková Dušátková, Stano Pekár, Ondřej Michálek, Eva Líznarová

& William O. C. Symondson

**Supplementary Information**

Table S1: Prey and predator sequences detected in *Callilepis nocturna* ……………………………………..2

Table S2: Prey and predator sequences detected in *Callilepis schuzsteri* ………………………………..….7

Table S3: Prey and predator sequences detected in *Nomisia exornata ……………………..….……..….* 17

Table S4: The results of primers laboratory tests with different annealing temperatures ………… 31

**Table S1.** List of DNA sequences of prey and the predator detected from the gut of *Callilepis nocturna*. **Total** = number of sequences without stop codons and reading frame shifts, appearing more than once; **ant primers** = MOTUs (= molecular operational taxonomic units) representing prey sequences amplified with ant specific primers; **ZBJ primers** = MOTUs representing other prey sequences amplified with general invertebrate primers (Zeale et al. 2011); **# seq.** = number of prey sequences in each MOTU per individual; **# predator seq.** = number of predator sequences. MOTUs represented by less than 0.005% of the total number of valid sequences are in grey, MOTUs represented by less than 0.5% of the valid sequences obtained from an individual spider (for ant prey only) are in blue. If the similarity was lower than 90%, prey was assigned only to a family level. Non-prey sequences (bacteria) are in violet. Sex/stage: F = adult female, M = adult male, J = juvenile.

| **Sex**  **/**  **ind.** | **Total** | **Prey** | | | | **# predator seq.** |
| --- | --- | --- | --- | --- | --- | --- |
|  |  | **Ant primers** | **# seq.** | **ZBJ primers** | **# seq.** |  |
| F1 | 8011 | *Formica*  *Formicidae unident.*  *Temnothorax*  *Temnothorax*  Formicidae unident. | \| 11 \| \| --- \| \| 2 \| \| 2 \| \| 2 \| \| 2 \| | ------- | ------- | 7992 |
| F2 | 3533 | *Lasius*  *Formicidae unident.*  *Lasius*  *Formica*  *Temnothorax*  *Lasius*  Formicidae unident.  Formicidae unident.  *Temnothorax*  *Lasius*  *Temnothorax*  Formicidae unident.  *Myrmica*  Formicidae unident.  *Dolichoderus* | \| 1490 \| \| --- \| \| 228 \| \| 100 \| \| 60 \| \| 7 \| \| 4 \| \| 4 \| \| 3 \| \| 3 \| \| 2 \| \| 2 \| \| 2 \| \| 2 \| \| 2 \| \| 2 \| | ------- | ------- | 1622 |
| J3 | 23156 | *Lasius*  *Messor*  *Formica*  Proteobacteria  Messor | \| 23042 \| \| --- \| \| 86 \| \| 23 \| \| 3 \| \| 2 \| | ------- | ------- | ------- |
| J4 | 101501 | *Camponotus*  Formicidae unident.  *Lasius*  *Camponotus*  *Formica*  *Myrmica*  *Lasius*  *Temnothorax*  *Lasius*  Proteobacteria  *Lasius*  Formicidae unident.  Formicidae unident. | \| 83474 \| \| --- \| \| 10014 \| \| 844 \| \| 152 \| \| 97 \| \| 13 \| \| 2 \| \| 2 \| \| 2 \| \| 3 \| \| 15 \| \| 11 \| \| 2 \| | Diptera: Heleomyzidae | 2 | 6868 |
| F5 | 4094 | ------- | ------- | ------- | ------- | 4094 |
| F6 | 474 | *Lasius*  Formicidae unident.  Formicidae unident.  *Camponotus*  *Formica*  Formicidae unident.  Formicidae unident.  Formicidae unident. | \| 155 \| \| --- \| \| 147 \| \| 137 \| \| 21 \| \| 6 \| \| 4 \| \| 2 \| \| 2 \| | ------- | ------- | ------- |
| F7 | 1358 | ------- | ------- | ------- | ------- | 1358 |
| M8 | 1549 | *Formica*  Formicidae unident. | \| 6 \| \| --- \| \| 3 \| | ------- | ------- | 1540 |
| F9 | 4165 | *Formica*  *Formica* | \| 4 \| \| --- \| \| 2 \| | ------- | ------- | 4159 |
| M10 | 17551 | *Formica*  *Lasius*  Formicidae unident.  Formicidae unident.  Formicidae unident.  Formicidae unident.  *Lasius*  *Lasius*  *Formica* | \| 17311 \| \| --- \| \| 102 \| \| 62 \| \| 61 \| \| 7 \| \| 2 \| \| 2 \| \| 2 \| \| 2 \| | ------- | ------- | ------- |
| F11 | 1407 | *Camponotus*  *Formica*  *Lasius* | \| 27 \| \| --- \| \| 2 \| \| 2 \| | ------- | ------- | 1376 |
| F12 | 1756 | Formicidae unident.  *Camponotus*  *Formica*  *Lasius* | \| 113 \| \| --- \| \| 15 \| \| 14 \| \| 2 \| | ------- | ------- | 1612 |
| F13 | 15163 | *Temnothorax*  *Formica*  *Temnothorax*  *Lasius*  *Lasius*  Formicidae unident. | \| 24 \| \| --- \| \| 20 \| \| 6 \| \| 2 \| \| 2 \| \| 6 \| | ------- | ------- | 15103 |
| J14 | 93596 | *Lasius*  *Tetramorium*  *Formica*  *Temnothorax*  Formicidae unident.  Formicidae unident.  *Dolichoderus*  Formicidae unident.  *Lasius*  Formicidae unident.  Proteobacteria  *Tetramorium* | \| 93129 \| \| --- \| \| 380 \| \| 55 \| \| 6 \| \| 2 \| \| 2 \| \| 2 \| \| 2 \| \| 2 \| \| 2 \| \| 7 \| \| 7 \| | ------- | ------- | ------- |
| M15 | 21978 | *Lasius*  *Formica*  *Lasius*  Formicidae unident.  *Formica*  Proteobacteria | \| 16405 \| \| --- \| \| 7 \| \| 2 \| \| 2 \| \| 2 \| \| 10 \| | ------- | ------- | 5550 |
| F16 | 5018 | Formicidae unident.  *Camponotus*  *Lasius*  *Formica*  *Myrmica*  *Lasius*  *Camponotus*  *Formica*  *Temnothorax*  Formicidae unident.  *Temnothorax*  Formicidae unident.  *Camponotus* | \| 629 \| \| --- \| \| 590 \| \| 107 \| \| 40 \| \| 2 \| \| 2 \| \| 2 \| \| 2 \| \| 2 \| \| 2 \| \| 2 \| \| 3 \| \| 2 \| | ------- | ------- | 3633 |
| J17 | 2249 | Formicidae unident.  *Formica* | \| 2 \| \| --- \| \| 2 \| | ------- | ------- | 2245 |
| J18 | 5704 | *Lasius*  *Formica*  *Lasius*  *Temnothorax* | \| 5694 \| \| --- \| \| 6 \| \| 2 \| \| 2 \| | ------- | ------- | ------- |
| J19 | 789 | Formicidae unident.  Formicidae unident.  *Formica*  *Lasius*  Proteobacteria | \| 52 \| \| --- \| \| 2 \| \| 2 \| \| 2 \| \| 2 \| | ------- | ------- | 729 |
| F20 | 4016 | *Messor*  *Aphaenogaster*  *Formica*  Formicidae unident.  *Lasius*  *Lasius*  *Aphaenogaster* | \| 1013 \| \| --- \| \| 1146 \| \| 15 \| \| 8 \| \| 4 \| \| 2 \| \| 2 \| | ------- | ------- | 1826 |
| J21 | 37400 | *Formica*  *Temnothorax*  Formicidae unident.  *Formica*  Formicidae unident.  *Messor* | \| 23923 \| \| --- \| \| 1435 \| \| 88 \| \| 3 \| \| 2 \| \| 2 \| | ------- | ------- | 11947 |
| J22 | 100439 | *Formica*  *Plagiolepis*  *Temnothorax*  *Lasius*  *Lasius*  *Formica*  *Temnothorax*  *Temnothorax*  *Lasius*  *Lasius*  *Lasius*  *Lasius*  Formicidae unident.  *Messor*  Formicidae unident. | \| 94194 \| \| --- \| \| 4794 \| \| 1062 \| \| 250 \| \| 93 \| \| 13 \| \| 9 \| \| 4 \| \| 2 \| \| 2 \| \| 2 \| \| 2 \| \| 2 \| \| 2 \| \| 8 \| | ------- | ------- | ------- |
| J23 | 41129 | *Lasius*  *Plagiolepis*  *Formica*  *Formica*  *Formica* | \| 31809 \| \| --- \| \| 5217 \| \| 22 \| \| 2 \| \| 2 \| | ------- | ------- | 4077 |
| J24 | 8842 | *Camponotus*  Formicidae unident.  Formicidae unident.  *Formica*  *Lasius*  *Lasius*  *Plagiolepis*  *Formica*  *Temnothorax*  *Formica*  *Plagiolepis*  *Temnothorax*  Formicidae unident.  Formicidae unident.  Formicidae unident.  *Formica*  Formicidae unident.  Formicidae unident. | \| 372 \| \| --- \| \| 272 \| \| 72 \| \| 103 \| \| 42 \| \| 38 \| \| 28 \| \| 17 \| \| 14 \| \| 10 \| \| 2 \| \| 2 \| \| 2 \| \| 2 \| \| 2 \| \| 2 \| \| 2 \| \| 2 \| | ------- | ------- | 7858 |
| Total | 504,878 | 421,287 | | 2 | | 83,589 |

**Table S2.** List of DNA sequences of prey and the predator detected from the gut of *Callilepis schuzsteri*. **Total** = number of sequences without stop codons and reading frame shifts, appearing more than once; **ant primers** = MOTUs (= molecular operational taxonomic units) representing prey sequences amplified with ant specific primers; **ZBJ primers** = MOTUs representing other prey sequences amplified with general invertebrate primers (Zeale et al. 2011); **# seq.** = number of prey sequences in each MOTU per individual; **# predator seq.** = number of predator sequences. MOTUs represented by less than 0.005% of the total number of valid sequences are in grey, MOTUs represented by less than 0.5% of the valid sequences obtained from an individual spider (for ant prey only) are in blue. Sequences with more than 98% similarity with databases have a species stated in brackets. If the similarity was lower than 90%, prey was assigned only to a family level. Non-prey sequences (bacteria) are in violet. Sex/stage: F = adult female, M = adult male, J = juvenile.

| **Sex**  **/**  **ind.** | **Total** | Prey | | | | **# predator seq.** |
| --- | --- | --- | --- | --- | --- | --- |
|  |  | **Ant primers** | **# seq.** | **ZBJ primers** | **# seq.** |  |
| F1 | 6255 | *Formica*  *Formica*  *Formica*  *Formica*  *Temnothorax* | \| 55 \| \| --- \| \| 3 \| \| 2 \| \| 2 \| \| 2 \| | ------- | ------- | 6191 |
| F3 | 2841 | *Formica*  *Lasius*  Proteobacteria | \| 28 \| \| --- \| \| 2 \| \| 5 \| | ------- | ------- | 2806 |
| F4 | 41437 | *Formica*  *Camponotus*  Formicidae unident.  *Formica*  *Myrmica*  *Camponotus*  *Aphaenogaster*  *Lasius*  Formicidae unident.  *Formica*  Formicidae unident.  *Formica*  Formicidae unident. | \| 36047 \| \| --- \| \| 2254 \| \| 2204 \| \| 842 \| \| 27 \| \| 13 \| \| 2 \| \| 2 \| \| 2 \| \| 9 \| \| 4 \| \| 3 \| \| 2 \| | Diptera | 2 | 24 |
| F6 | 267 | *Formica*  *Lasius*  *Lasius*  Proteobacteria | \| 115 \| \| --- \| \| 118 \| \| 26 \| \| 8 \| | ------- | ------- | ------- |
| F7 | 42621 | *Formica*  Proteobacteria  *Formica*  *Lasius*  *Lasius*  Proteobacteria | \| 26091 \| \| --- \| \| 16091 \| \| 193 \| \| 232 \| \| 6 \| \| 2 \| | ------- | ------- | 6 |
| F8 | 41670 | *Formica*  *Formica*  *Lasius*  *Lasius*  *Camponotus*  Formicidae unident. Proteobacteria | \| 39944 \| \| --- \| \| 1045 \| \| 20 \| \| 3 \| \| 2 \| \| 2 \| \| 7 \| | Insecta unident. | 490 | 157 |
| F9 | 5057 | *Formica*  *Lasius*  *Camponotus*  *Myrmica*  *Dolichoderus*  *Formica*  Formicidae unident.  *Formica*  Proteobacteria | \| 3576 \| \| --- \| \| 1428 \| \| 18 \| \| 17 \| \| 6 \| \| 4 \| \| 2 \| \| 2 \| \| 4 \| | ------- | ------- | ------- |
| F10 | 3346 | *Formica*  *Formica*  *Lasius* | \| 3325 \| \| --- \| \| 16 \| \| 5 \| | ------- | ------- | ------- |
| F11 | 2916 | *Formica*  *Formica*  *Lasius* | \| 1979 \| \| --- \| \| 920 \| \| 17 \| | ------- | ------- | ------- |
| F12 | 6711 | *Formica*  *Formica*  *Lasius*  *Myrmica*  *Formica*  *Lasius*  Formicidae unident.  *Formica*  Proteobacteria | \| 6548 \| \| --- \| \| 74 \| \| 20 \| \| 4 \| \| 2 \| \| 2 \| \| 2 \| \| 4 \| \| 57 \| | ------- | ------- | ------- |
| F13 | 1930 | *Formica*  *Myrmica*  Formicidae unident.  *Formica*  *Lasius*  *Temnothorax*  Formicidae unident.  Formicidae unident. | \| 1616 \| \| --- \| \| 226 \| \| 69 \| \| 9 \| \| 4 \| \| 2 \| \| 2 \| \| 2 \| | ------- | ------- | ------- |
| F14 | 11537 | *Formica*  *Lasius*  *Formica*  *Formica*  *Plagiolepis*  *Formica*  *Temnothorax*  Formicidae unident.  *Formica*  *Lasius*  Proteobacteria  Formicidae unident. | \| 7167 \| \| --- \| \| 4243 \| \| 55 \| \| 23 \| \| 10 \| \| 3 \| \| 3 \| \| 3 \| \| 2 \| \| 2 \| \| 24 \| \| 2 \| | -------- | ------- | ------- |
| F15 | 10844 | *Lasius*  *Formica*  *Formica*  *Myrmica*  *Camponotus*  *Formica*  Proteobacteria  *Lasius* | \| 5841 \| \| --- \| \| 4883 \| \| 28 \| \| 14 \| \| 11 \| \| 2 \| \| 2 \| \| 3 \| | ------- | ------- | 60 |
| F16 | 22147 | *Formica*  *Lasius*  *Formica*  *Lasius*  Proteobacteria | \| 20671 \| \| --- \| \| 858 \| \| 2 \| \| 2 \| \| 10 \| | Insecta unident. | 4 | 600 |
| F17 | 20397 | *Dolichoderus*  *Lasius*  *Camponotus*  *Lasius*  *Formica*  Formicidae unident.  *Lasius*  Formicidae unident.  *Myrmica* Proteobacteria  Formicidae unident.  *Dolichoderus*  Formicidae unident.  Formicidae unident. | \| 13111 \| \| --- \| \| 5332 \| \| 1313 \| \| 370 \| \| 99 \| \| 36 \| \| 30 \| \| 3 \| \| 4 \| \| 24 \| \| 5 \| \| 4 \| \| 3 \| \| 2 \| | Camponotus | 61 | ------- |
| F18 | 1735 | *Formica*  *Lasius*  *Aphaenogaster*  *Lasius* | \| 1700 \| \| --- \| \| 27 \| \| 6 \| \| 2 \| | ------- | ------- | ------- |
| F19 | 3954 | *Formica*  *Lasius*  *Aphaenogaster*  *Lasius*  *Formica* | \| 3444 \| \| --- \| \| 470 \| \| 18 \| \| 14 \| \| 8 \| | ------- | ------- | ------- |
| F22 | 15495 | *Formica*  *Lasius*  *Formica*  *Dolichoderus*  *Lasius*  *Plagiolepis*  *Aphaenogaster*  *Lasius*  Formicidae unident. | \| 13537 \| \| --- \| \| 281 \| \| 11 \| \| 6 \| \| 6 \| \| 4 \| \| 2 \| \| 2 \| \| 2 \| | ------- | ------- | 1644 |
| M1 | 7824 | *Formica*  *Camponotus*  Formicidae unident.  Proteobacteria  *Formica* | \| 6484 \| \| --- \| \| 866 \| \| 57 \| \| 320 \| \| 10 \| | Diptera | 23 | 64 |
| M2 | 8444 | *Lasius*  *Formica*  Formicidae unident.  *Temnothorax*  *Formica*  Proteobacteria  *Temnothorax*  *Lasius*  Formicidae unident. | \| 7319 \| \| --- \| \| 21 \| \| 11 \| \| 2 \| \| 2 \| \| 1073 \| \| 10 \| \| 4 \| \| 2 \| | ------- | ------- | ------- |
| M3 | 21539 | *Formica*  *Myrmica*  *Temnothorax*  *Formica*  Formicidae unident.  *Lasius*  *Lasius*  *Formica*  *Myrmica*  Formicidae unident.  Proteobacteria *Temnothorax*  *Temnothorax*  *Temnothorax* | \| 10047 \| \| --- \| \| 10995 \| \| 348 \| \| 17 \| \| 7 \| \| 5 \| \| 4 \| \| 3 \| \| 2 \| \| 2 \| \| 14 \| \| 6 \| \| 3 \| \| 2 \| | Diptera: Heleomyzidae | 84 | ------- |
| M4 | 6448 | *Formica*  *Temnothorax*  *Temnothorax* | \| 5329 \| \| --- \| \| 9 \| \| 2 \| | ------- | ------- | 1108 |
| M5 | 6832 | *Formica*  *Formica*  *Temnothorax* Formicidae unident.  *Formica* | \| 6791 \| \| --- \| \| 27 \| \| 7 \| \| 4 \| \| 3 \| | ------- | ------- | 1094 |
| M6 | 38441 | *Formica*  *Temnothorax*  *Lasius*  *Temnothorax*  *Lasius*  Formicidae unident.  *Myrmica*  *Formica*  Proteobacteria  Formicidae unident. | \| 37589 \| \| --- \| \| 691 \| \| 36 \| \| 31 \| \| 23 \| \| 19 \| \| 9 \| \| 2 \| \| 26 \| \| 3 \| | Diptera | 2 | 10 |
| M7 | 1731 | *Formica*  *Lasius*  *Lasius*  *Temnothorax*  *Temnothorax* Proteobacteria  *Lasius* | \| 877 \| \| --- \| \| 356 \| \| 51 \| \| 2 \| \| 2 \| \| 441 \| \| 2 \| | ------- | ------- | ------- |
| M8 | 35124 | *Lasius*  *Myrmica*  *Lasius*  *Camponotus*  *Formica*  *Formica*  *Plagiolepis*  *Temnothorax*  Formicidae unident.  *Lasius*  *Formica*  Proteobacteria  Formicidae unident.  Formicidae unident.  Formicidae unident.  Formicidae unident. | \| 24781 \| \| --- \| \| 3184 \| \| 2841 \| \| 518 \| \| 189 \| \| 7 \| \| 6 \| \| 4 \| \| 3 \| \| 3 \| \| 2 \| \| 2 \| \| 7 \| \| 4 \| \| 2 \| \| 2 \| | ------- | ------- | 3569 |
| M9 | 8890 | *Formica*  Formicidae unident.  *Formica*  *Camponotus*  *Myrmica*  *Formica* (*F.pratensis*) Proteobacteria  *Myrmica* | \| 8385 \| \| --- \| \| 369 \| \| 108 \| \| 9 \| \| 7 \| \| 4 \| \| 6 \| \| 2 \| | ------- | ------- | ------- |
| M11 | 10775 | *Formica*  *Lasius*  *Myrmica*  *Lasius*  *Lasius*  *Lasius*  Proteobacteria | \| 9080 \| \| --- \| \| 1173 \| \| 150 \| \| 52 \| \| 4 \| \| 2 \| \| 6 \| | ------- | ------- | 308 |
| M12 | 9206 | *Lasius*  *Formica*  *Lasius*  *Formica*  *Lasius* | \| 5099 \| \| --- \| \| 2910 \| \| 1191 \| \| 4 \| \| 2 \| | ------- | ------- | ------- |
| M13 | 10863 | *Lasius*  *Lasius*  *Formica*  *Formica*  *Formica*  *Temnothorax*  Formicidae unident.  *Temnothorax*  *Lasius*  *Temnothorax* | \| 7186 \| \| --- \| \| 1793 \| \| 1855 \| \| 14 \| \| 3 \| \| 2 \| \| 2 \| \| 2 \| \| 2 \| \| 4 \| | ------- | ------- | ------- |
| M14 | 30083 | *Myrmica*  *Lasius*  *Lasius*  Formicidae unident.  *Temnothorax*  *Formica*  *Temnothorax*  *Temnothorax*  *Tetramorium*  *Formica*  Proteobacteria  Formicidae unident. | \| 24255 \| \| --- \| \| 2969 \| \| 2085 \| \| 294 \| \| 240 \| \| 113 \| \| 97 \| \| 6 \| \| 2 \| \| 2 \| \| 18 \| \| 2 \| | ------- | ------- | ------- |
| M15 | 165 | - | - | ------- | ------- | 165 |
| M16 | 11601 | *Formica*  *Lasius*  *Temnothorax*  Formicidae unident.  *Lasius*  *Myrmica*  *Temnothorax*  *Temnothorax*  *Plagiolepis*  *Formica*  *Temnothorax*  *Temnothorax*  Formicidae unident.  *Temnothorax*  *Camponotus*  *Lasius*  *Camponotus*  Formicidae unident. Proteobacteria  Formicidae unident. | \| 4728 \| \| --- \| \| 3399 \| \| 793 \| \| 471 \| \| 171 \| \| 69 \| \| 59 \| \| 17 \| \| 7 \| \| 4 \| \| 4 \| \| 3 \| \| 3 \| \| 3 \| \| 2 \| \| 2 \| \| 2 \| \| 2 \| \| 141 \| \| 2 \| | ------- | ------- | 1719 |
| M18 | 13345 | *Formica*  *Lasius*  *Lasius*  *Lasius*  *Myrmica*  *Aphaenogaster* Proteobacteria | \| 9633 \| \| --- \| \| 2837 \| \| 852 \| \| 4 \| \| 2 \| \| 2 \| \| 15 \| | ------- | ------- | ------- |
| M19 | 31688 | *Formica*  *Lasius*  *Formica*  *Myrmica*  *Dolichoderus*  *Lasius*  *Camponotus*  *Temnothorax* Proteobacteria | \| 22384 \| \| --- \| \| 108 \| \| 79 \| \| 43 \| \| 17 \| \| 2 \| \| 2 \| \| 2  9051 \| | ------- | ------- | ------- |
| M20 | 7561 | *Lasius*  *Formica*  Formicidae unident.  *Aphaenogaster* | \| 4503 \| \| --- \| \| 3023 \| \| 33 \| \| 2 \| | ------- | ------- | ------- |
| M22 | 55299 | *Myrmica*  *Lasius*  *Dolichoderus*  *Formica*  Formicidae unident.  Formica  Formicidae unident. Proteobacteria  Formicidae unident.  Formicidae unident.  Dolichoderus  Dolichoderus  Formicidae unident. | \| 7195 \| \| --- \| \| 2348 \| \| 1186 \| \| 780 \| \| 3 \| \| 2 \| \| 2 \| \| 43769  5 \| \| 3 \| \| 2 \| \| 2 \| \| 2 \| | ------- | ------- | ------- |
| M24 | 21641 | *Formica*  *Lasius*  *Formica*  *Lasius*  *Formica*  *Myrmica*  *Messor*  Formicidae unident.  *Myrmica* Proteobacteria  *Aphaenogaster*  *Lasius* | \| 10039 \| \| --- \| \| 8413 \| \| 3151 \| \| 11 \| \| 8 \| \| 7 \| \| 2 \| \| 2 \| \| 2 \| \| 2 \| \| 2 \| \| 2 \| | ------- | ------- | ------- |
| M25 | 39643 | *Formica*  *Myrmica*  *Formica*  *Formica*  *Lasius*  Formicidae unident. Proteobacteria | \| 28200 \| \| --- \| \| 3948 \| \| 31 \| \| 4 \| \| 2 \| \| 4 \| \| 14 \| | ------- | ------- | 7440 |
| M26 | 50129 | *Lasius*  *Lasius*  *Temnothorax*  *Temnothorax*  *Formica*  *Lasius*  *Formica*  Proteobacteria  *Temnothorax*  *Temnothorax*  Formicidae unident.  Formicidae unident. | \| 49445 \| \| --- \| \| 360 \| \| 45 \| \| 113 \| \| 56 \| \| 2 \| \| 2 \| \| 83 \| \| 17 \| \| 2 \| \| 2 \| \| 2 \| | ------- | ------- | ------- |
| M30 | 5235 | *Temnothorax*  Formicidae unident.  *Temnothorax*  *Temnothorax*  *Lasius*  *Formica*  *Aphaenogaster*  Proteobacteria  *Temnothorax* | \| 3345 \| \| --- \| \| 1191 \| \| 390 \| \| 182 \| \| 59 \| \| 25 \| \| 22 \| \| 4 \| \| 17 \| | ------- | ------- | ------- |
| Total | 674,763 | 647,132 | | 666 | | 26,965 |

**Table S3.** List of DNA sequences of prey and the predator detected from the gut of *Nomisia exornata*. **Total** = number of sequences without stop codons and reading frame shifts, appearing more than once; **ant primers** = MOTUs (= molecular operational taxonomic units) representing prey sequences amplified with ant specific primers; **ZBJ primers** = MOTUs representing other prey sequences amplified with general invertebrate primers (Zeale et al. 2011); **# seq.** = number of prey sequences in each MOTU per individual; **# predator seq.** = number of predator sequences. MOTUs represented by less than 0.005% of the total number of valid sequences are in grey, MOTUs represented by less than 0.5% of the valid sequences obtained from an individual spider (for ant prey only) are in blue. Sequences with more than 98% similarity with databases have a species stated in brackets. If the similarity was lower than 90%, prey was assigned only to a family level. Sex/stage: F = adult female, M = adult male, J = juvenile.

| **Sex**  **(stage)/**  **ind.** | **Total** | Prey | | | | **# predator seq.** |
| --- | --- | --- | --- | --- | --- | --- |
|  |  | **Ant primers** | **# seq.** | **ZBJ primers** | **# seq.** |  |
| M1 | 1864 | *Messor* (*M. barbarus*)  *Cataglyphis*  *Cataglyphis*  *Messor*  *Aphaenogaster* | 1036  367  162  79  6 | Arthropoda unident. | 21 | 193 |
| M2 | 2758 | *Messor* (*M. barbarus*)  *Lasius*  *Messor*  *Tetramorium*  Formicidae unident.  *Cataglyphis*  Dolichoderinae  *Aphaenogaster*  *Cataglyphis*  *Tetramorium*  Formicidae unident.  Formicidae unident.  *Tetramorium*  *Tetramorium*  Formicidae unident. | 2337  110  102  64  41  34  22  19  10  9  2  2  2  2  2 | ------- | ------- | ------- |
| M3 | 9834 | *Messor* (*M. barbarus*)  *Messor*  Dolichoderinae  Formicidae unident. | 9373  4  2  2 | Arthropoda unident. | 12 | 441 |
| M4 | 11325 | *Messor* (*M. barbarus*)  *Messor*  *Messor*  *Cataglyphis*  *Tetramorium* | 6546  4665  12  2  2 | ------ | ------ | 98 |
| F1 | 14899 | *Tetramorium*  *Plagiolepis*  *Messor* (*M. barbarus*)  *Messor*  *Aphaenogaster*  *Aphaenogaster*  Formicidae unident.  Formicidae unident. | 14509  136  71  28  5  3  2  2 | Lepidoptera | 4 | 139 |
| F2 | 18730 | *Tetramorium*  Formicidae unident.  *Messor* (*M. barbarus*)  *Messor*  Formicidae unident.  *Plagiolepis*  Formicidae unident.  *Lasius*  *Aphaenogaster*  *Plagiolepis*  Dolichoderinae  Formicidae unident.  *Tetramorium*  Formicidae unident.  *Tetramorium*  Formicidae unident.  Formicidae unident.  *Aphaenogaster*  Formicidae unident. | 10730  5258  1469  479  223  131  89  81  75  18  10  9  4  2  2  4  4  2  2 | ------ | ------ | 140 |
| F3 | 58824 | *Messor* (*M. barbarus*)  *Messor*  *Messor*  *Tetramorium*  *Aphaenogaster* | \| 27911 \| \| --- \| \| 3423 \| \| 18 \| \| 13 \| \| 2 \| | Lepidoptera | 27446 | 11 |
| F4 | 33558 | *Messor* (*M. barbarus*)  *Messor*  *Tetramorium*  *Tetramorium*  *Formicidae unident.*  *Aphaenogaster*  Formicidae unident.  *Messor*  *Messor* | \| 26742 \| \| --- \| \| 5863 \| \| 634 \| \| 75 \| \| 16 \| \| 10 \| \| 2 \| \| 2 \| \| 2 \| | Lepidoptera  Lepidoptera | 6  2 | 204 |
| F5 | 11656 | *Messor* (*M. barbarus*)  *Messor*  *Aphaenogaster*  Formicidae unident. *Tetramorium* (*T. forte*)  *Tetramorium*  *Messor*  *Plagiolepis* | \| 4736 \| \| --- \| \| 3887 \| \| 1424 \| \| 575 \| \| 36 \| \| 26 \| \| 8 \| \| 2 \|   2 | Lepidoptera | 2 | 960 |
| F6 | 29640 | *Tetramorium*  *Aphaenogaster*  *Messor* (*M. barbarus*)  *Messor*  Formicidae unident.  *Tetramorium*  Formicidae unident.  *Lasius*  *Tetramorium*  *Tetramorium*  *Tetramorium* (*T. forte*)  *Plagiolepis*  Formicidae unident.  *Tapinoma*  Formicidae unident.  *Messor*  Dolichoderinae  Formicidae unident. | \| 17833 \| \| --- \| \| 6542 \| \| 1634 \| \| 953 \| \| 608 \| \| 219 \| \| 131 \| \| 109 \| \| 20 \| \| 51 \| \| 12 \| \| 12 \| \| 9 \| \| 4 \| \| 4 \| \| 12 \| \| 2 \| \| 4 \| | Arthropoda unident. | 2 | 1479 |
| F7 | 1461 | *Messor* (*M. barbarus*)  *Tetramorium*  *Messor* | \| 84 \| \| --- \| \| 10 \| \| 21 \| | Arthropoda unident. Lepidoptera | 11  2 | 1333 |
| F8 | 38188 | *Tetramorium*  *Messor* (*M. barbarus*)  *Messor*  *Plagiolepis*  *Aphaenogaster*  *Tapinoma*  Formicidae unident. | \| 36897 \| \| --- \| \| 286 \| \| 31 \| \| 2 \| \| 2 \| \| 2 \| \| 2 \| | Lepidoptera | 3 | 963 |
| F9 | 25603 | Messor (M. barbarus)  Messor  Plagiolepis  Tetramorium  Aphaenogaster  Formicidae unident.  Messor  Formicidae unident.  Formicidae unident. | \| 19200 \| \| --- \| \| 4204 \| \| 584 \| \| 363 \| \| 113 \| \| 29 \| \| 2 \| \| 4 \| \| 2 \| | Arthropoda unident. | 4 | 1098 |
| F10 | 11074 | *Messor* (*M. barbarus*)  *Messor*  *Tetramorium*  *Formicidae unident.*  *Lasius*  Formicidae unident.  *Messor*  *Plagiolepis*  Dolichoderinae  *Tapinoma*  Formicidae unident.  Formicidae unident.  *Tetramorium*  Formicidae unident. | \| 3201 \| \| --- \| \| 2933 \| \| 2378 \| \| 187 \| \| 142 \| \| 71 \| \| 24 \| \| 23 \| \| 9 \| \| 8 \| \| 6 \| \| 4 \| \| 2 \| \| 2 \| | Lepidoptera  Arthropoda unident. | 27  4 | 2048 |
| F11 | 38505 | *Messor* (*M. barbarus*)  *Messor*  *Tetramorium*  Formicidae unident. | \| 30129 \| \| --- \| \| 3644 \| \| 95 \| \| 6 \| | Arthropoda unident. Diptera: Cecidomyiidae | 4  2 | 4625 |
| F12 | 1802 | *Messor* (*M. barbarus*)  *Tetramorium*  *Messor* | \| 164 \| \| --- \| \| 144 \| \| 45 \| | Arthropoda unident. Lepidoptera | 2  2 | 1445 |
| F13 | 19774 | *Messor* (*M. barbarus*)  *Plagiolepis*  *Tetramorium*  *Messor*  *Aphaenogaster* | \| 12921 \| \| --- \| \| 3579 \| \| 2541 \| \| 59 \| \| 5 \| | Araneae unident. | 10 | 659 |
| F14 | 14449 | *Messor* (*M. barbarus*)  *Lasius*  *Tetramorium*  *Messor*  *Tetramorium*  *Plagiolepis*  Formicidae unident.  Formicidae unident.  Formicidae unident.  Formicidae unident.  *Lasius*  *Lasius*  *Lasius*  Formicidae unident.  Formicidae unident. | \| 5248 \| \| --- \| \| 5054 \| \| 1920 \| \| 429 \| \| 260 \| \| 84 \| \| 60 \| \| 8 \| \| 8 \| \| 2 \| \| 13 \| \| 12 \| \| 2 \| \| 2 \| \| 2 \| | Arthropoda unident. | 39 | 1312 |
| J1 | 4441 | *Messor* (*M. barbarus*)  *Tetramorium*  *Messor* | \| 126 \| \| --- \| \| 68 \| \| 11 \| | ------ | ------ | 4236 |
| J2 | 74993 | *Tetramorium*  *Messor* (*M. barbarus*)  *Plagiolepis*  *Formica* | \| 72408 \| \| --- \| \| 130 \| \| 33 \| \| 26 \| | Insecta unident. | 1462 | 934 |
| J3 | 6897 | *Messor* (*M. barbarus*)  *Tetramorium*  *Messor*  *Plagiolepis* | \| 5410 \| \| --- \| \| 531 \| \| 445 \| \| 161 \| | ------ | ------ | 350 |
| J4 | 7517 | *Messor* (*M. barbarus*)  *Tetramorium*  *Messor*  *Tetramorium*  *Lasius*  *Plagiolepis*  Formicidae unident.  Formicidae unident.  *Messor*  *Messor* | \| 4238 \| \| --- \| \| 1455 \| \| 897 \| \| 305 \| \| 14 \| \| 9 \| \| 6 \| \| 6 \| \| 2 \| \| 2 \| | ------ | ------ | 583 |
| J5 | 7977 | *Tetramorium*  *Messor* (*M. barbarus*) | \| 7020 \| \| --- \| \| 28 \| | ------- | ------ | 929 |
| J6 | 17979 | Messor (M. barbarus)  Tetramorium  Messor  Plagiolepis  Formicidae unident. | \| 14054 \| \| --- \| \| 1563 \| \| 1839 \| \| 117 \| \| 2 \| | ------- | ------ | 404 |
| J7 | 10939 | *Messor* (*M. barbarus*)  *Tetramorium*  *Messor*  Formicidae unident.  *Aphaenogaster*  *Plagiolepis* | \| 6706 \| \| --- \| \| 2691 \| \| 790 \| \| 232 \| \| 96 \| \| 61 \| | ------- | ------ | 363 |
| J8 | 24367 | *Messor* (*M. barbarus*)  *Tetramorium*  *Tetramorium*  *Messor*  *Lasius*  Formicidae unident.  Dolichoderinae  Formicidae unident.  Formicidae unident.  *Plagiolepis*  Crabronidae (*Diodontus*)  *Lasius*  Formicidae unident.  *Cataglyphis*  *Aphaenogaster* | \| 15817 \| \| --- \| \| 5034 \| \| 1752 \| \| 1319 \| \| 78 \| \| 28 \| \| 12 \| \| 10 \| \| 10 \| \| 6 \| \| 6 \| \| 2 \| \| 2 \| \| 2 \| \| 2 \| | ------- | ------ | 287 |
| J9 | 1156 | *Messor* (*M. barbarus*)  *Messor* | \| 44 \| \| --- \| \| 7 \| | ------- | ------ | 1105 |
| J10 | 18731 | *Messor* (*M. barbarus*)  Crabronidae (*Diodontus*)  *Tetramorium*  *Messor*  *Tetramorium* | \| 11839 \| \| --- \| \| 5410 \| \| 552 \| \| 517 \| \| 2 \| | Araneae unident. | 6 | 405 |
| J11 | 23133 | *Tetramorium*  *Messor*  *Messor* (*M. barbarus*)  *Messor* | \| 8807 \| \| --- \| \| 8336 \| \| 4882 \| \| 37 \| | Lepidoptera | 4 | 1067 |
| J12 | 71453 | *Tetramorium*  *Messor* (*M. barbarus*)  *Messor*  *Lasius*  Formicidae unident.  Formicidae unident.  Formicidae unident.  *Lasius*  Dolichoderinae  Formicidae unident.  Formicidae unident.  *Lasius*  Formicidae unident.  *Tetramorium*  Formicidae unident. | \| 53856 \| \| --- \| \| 10250 \| \| 3726 \| \| 156 \| \| 156 \| \| 70 \| \| 24 \| \| 10 \| \| 5 \| \| 4 \| \| 3 \| \| 2 \| \| 2 \| \| 5 \| \| 3 \| | ------- | ------ | 3181 |
| J13 | 897 | *Messor* (*M. barbarus*)  *Tetramorium* | \| 92 \| \| --- \| \| 35 \| | Lepidoptera  Araneae unident. | 4  2 | 764 |
| J14 | 63598 | *Messor*  *Tetramorium*  *Messor*  *Lasius*  *Messor* (*M. barbarus*)  Formicidae unident.  Formicidae unident. | \| 33024 \| \| --- \| \| 20028 \| \| 133 \| \| 113 \| \| 96 \| \| 2 \| \| 18 \| | Lepidoptera  Lepidoptera | 8068  16 | 2100 |
| J15 | 27078 | *Tetramorium*  *Plagiolepis*  *Messor* (*M. barbarus*)  *Tetramorium*  *Messor*  Dolichoderinae | \| 26511 \| \| --- \| \| 372 \| \| 78 \| \| 7 \| \| 2 \| \| 2 \| | ------- | ------ | 106 |
| J16 | 21923 | *Messor* (*M. barbarus*)  *Tetramorium*  *Messor*  Formicidae unident.  *Plagiolepis*  *Aphaenogaster* | \| 13460 \| \| --- \| \| 5321 \| \| 86 \| \| 83 \| \| 40 \| \| 2 \| | Lepidoptera  Insecta unident. Collembola  Insecta unident. | 2896  20  5  4 | 6 |
| J17 | 39732 | *Tetramorium*  *Messor* (*M. barbarus*)  *Plagiolepis*  *Messor* | \| 25786 \| \| --- \| \| 13459 \| \| 312 \| \| 123 \| | ------- | ------ | 52 |
| J18 | 16997 | *Tetramorium*  *Plagiolepis*  Formicidae unident.  *Messor*  Formicidae unident.  Formicidae unident. | \| 16570 \| \| --- \| \| 44 \| \| 14 \| \| 4 \| \| 21 \| \| 4 \| | Lepidoptera | 8 | 332 |
| J19 | 13849 | *Tetramorium*  *Plagiolepis*  *Messor* (*M. barbarus*)  *Messor* | \| 13344 \| \| --- \| \| 417 \| \| 44 \| \| 2 \| | ------- | ------ | 42 |
| J20 | 2600 | *Tetramorium*  *Tetramorium*  Formicidae unident.  *Messor*  *Plagiolepis*  Formicidae unident. | \| 1714 \| \| --- \| \| 564 \| \| 184 \| \| 85 \| \| 12 \| \| 2 \| | ------- | ------ | 39 |
| J21 | 3605 | *Tetramorium*  *Messor* (*M. barbarus*)  *Plagiolepis*  *Tetramorium* | \| 2416 \| \| --- \| \| 1004 \| \| 26 \| \| 11 \| | ------- | ------ | 148 |
| J22 | 3898 | *Messor* (*M. barbarus*)  *Tetramorium*  *Tetramorium*  *Plagiolepis*  *Messor*  *Formica* | \| 3280 \| \| --- \| \| 327 \| \| 75 \| \| 12 \| \| 2 \| \| 4 \| | ------- | ------ | 198 |
| J23 | 984 | *Tetramorium*  *Plagiolepis* | \| 913 \| \| --- \| \| 2 \| | ------- | ------ | 69 |
| J24 | 7151 | *Tetramorium*  *Messor*  *Plagiolepis*  *Messor* (*M. barbarus*) | \| 5912 \| \| --- \| \| 625 \| \| 408 \| \| 6 \| | ------- | ------ | 200 |
| J25 | 4145 | Dolichoderinae  *Tetramorium*  *Messor* (*M. barbarus*)  *Plagiolepis*  *Tetramorium*  *Messor*  Formicidae unident.  *Messor*  Formicidae unident. | \| 2714 \| \| --- \| \| 1228 \| \| 71 \| \| 47 \| \| 6 \| \| 4 \| \| 3 \| \| 2 \| \| 4 \| | Lepidoptera | 12 | 54 |
| J26 | 3770 | *Tetramorium*  *Messor* (*M. barbarus*)  *Plagiolepis*  Dolichoderinae  *Messor*  *Tetramorium*  Formicidae unident. | \| 1299 \| \| --- \| \| 1110 \| \| 1034 \| \| 248 \| \| 27 \| \| 26 \| \| 4 \| | ------- | ------ | 22 |
| J27 | 7217 | *Messor* (*M. barbarus*)  *Messor*  Dolichoderinae  *Tetramorium*  *Plagiolepis*  Formicidae unident.  *Tapinoma*  *Tapinoma* | \| 4171 \| \| --- \| \| 2251 \| \| 304 \| \| 251 \| \| 52 \| \| 9 \| \| 2 \| \| 2 \| | ------- | ------ | 175 |
| J28 | 4804 | *Tetramorium*  Dolichoderinae  *Plagiolepis*  Formicidae unident. | \| 2548 \| \| --- \| \| 1173 \| \| 939 \| \| 2 \| | Araneae unident. | 4 | 138 |
| J29 | 5833 | Dolichoderinae  *Tetramorium*  *Messor* (*M. barbarus*)  *Plagiolepis*  *Messor*  Formicidae unident. | \| 5184 \| \| --- \| \| 271 \| \| 17 \| \| 15 \| \| 8 \| \| 2 \| | Araneae unident. | 4 | 332 |
| J30 | 20171 | *Tetramorium*  *Messor* (*M. barbarus*)  Dolichoderinae  *Messor*  *Plagiolepis*  Formicidae unident.  Formicidae unident.  *Tetramorium*  *Tetramorium*  *Formica*  Formicidae unident. | \| 15775 \| \| --- \| \| 2881 \| \| 695 \| \| 327 \| \| 204 \| \| 13 \| \| 9 \| \| 2 \| \| 2 \| \| 2 \| \| 2 \| | Lepidoptera | 47 | 212 |
| J31 | 5789 | *Tetramorium*  *Tetramorium*  *Messor* (*M. barbarus*)  *Messor*  Dolichoderinae  *Plagiolepis*  Formicidae unident.  Formicidae unident.  Formicidae unident.  Formicidae unident. | \| 3037 \| \| --- \| \| 1532 \| \| 941 \| \| 48 \| \| 9 \| \| 2 \| \| 2 \| \| 2 \| \| 2 \| \| 2 \| | Lepidoptera | 20 | 192 |
| J32 | 26633 | *Messor* (*M. barbarus*)  *Tetramorium*  *Messor*  Formicidae unident.  Dolichoderinae  *Plagiolepis*  *Messor*  Formicidae unident. | \| 14290 \| \| --- \| \| 9116 \| \| 585 \| \| 556 \| \| 62 \| \| 20 \| \| 5 \| \| 2 \| | Lepidoptera | 4 | 1993 |
| J33 | 26386 | *Tetramorium*  Dolichoderinae | \| 23243 \| \| --- \| \| 174 \| | Lepidoptera  Insecta unident. | 2420  2 | 547 |
| J34 | 26030 | *Tetramorium*  *Messor* (*M. barbarus*)  Dolichoderinae  *Messor*  Formicidae unident.  Formicidae unident. | \| 16199 \| \| --- \| \| 4686 \| \| 711 \| \| 464 \| \| 48 \| \| 20 \| | Lepidoptera | 10 | 3892 |
| J35 | 1427 | *Messor* (*M. barbarus*)  *Tetramorium*  Dolichoderinae  *Plagiolepis*  *Messor* | \| 1012 \| \| --- \| \| 326 \| \| 50 \| \| 9 \| \| 4 \| | ------- | ------ | 26 |
| J36 | 958 | Formicidae unident.  *Messor* (*M. barbarus*)  *Tetramorium*  *Messor*  Dolichoderinae  *Cataglyphis*  Formicidae unident. | \| 630 \| \| --- \| \| 155 \| \| 118 \| \| 6 \| \| 4 \| \| 2 \| \| 2 \| | ------- | ------ | 41 |
| J37 | 722 | *Plagiolepis*  *Messor* (*M. barbarus*)  Dolichoderinae  Formicidae unident.  *Messor*  *Tetramorium* | \| 401 \| \| --- \| \| 46 \| \| 40 \| \| 7 \| \| 4 \| \| 2 \| | ------- | ------ | 222 |
| J38 | 1916 | *Tetramorium*  Dolichoderinae  *Plagiolepis*  Formicidae unident. | \| 1589 \| \| --- \| \| 184 \| \| 15 \| \| 4 \| | ------- | ------ | 124 |
| J39 | 5740 | Dolichoderinae  *Messor* (*M. barbarus*)  *Tetramorium*  *Messor*  Formicidae unident.  *Plagiolepis* | \| 3420 \| \| --- \| \| 1617 \| \| 316 \| \| 91 \| \| 34 \| \| 8 \| | ------- | ------ | 254 |
| J40 | 6971 | *Messor*  Dolichoderinae  *Messor* (*M. barbarus*)  *Plagiolepis*  *Tetramorium*  Formicidae unident.  Formicidae unident.  Formicidae unident.  Formicidae unident.  *Messor*  *Tetramorium*  Formicidae unident.  *Aphaenogaster* | \| 2933 \| \| --- \| \| 2382 \| \| 1187 \| \| 201 \| \| 146 \| \| 9 \| \| 7 \| \| 2 \| \| 2 \| \| 2 \| \| 2 \| \| 14 \| \| 2 \| | Lepidoptera | 2 | 79 |
| J41 | 37 | ------- | ------- | Lepidoptera | 2 | 35 |
| J42 | 9223 | *Messor* (*M. barbarus*)  *Aphaenogaster*  *Messor*  *Tetramorium*  *Plagiolepis*  Dolichoderinae  Formicidae unident.  Formicidae unident.  Formicidae unident.  Formicidae unident. | \| 4934 \| \| --- \| \| 2083 \| \| 1497 \| \| 360 \| \| 167 \| \| 13 \| \| 2 \| \| 2 \| \| 2 \| \| 2 \| | ------- | ------ | 161 |
| J43 | 12021 | *Tetramorium*  *Plagiolepis*  Dolichoderinae  *Aphaenogaster*  *Messor* | \| 10097 \| \| --- \| \| 129 \| \| 6 \| \| 4 \| \| 2 \| | Lepidoptera | 1596 | 187 |
| J44 | 14812 | *Tetramorium*  *Plagiolepis*  Dolichoderinae  *Messor* (*M. barbarus*)  *Messor*  *Aphaenogaster* | \| 13172 \| \| --- \| \| 835 \| \| 114 \| \| 70 \| \| 30 \| \| 2 \| | Lepidoptera | 4 | 585 |
| J45 | 13036 | *Messor* (*M. barbarus*)  *Tetramorium*  Formicidae unident.  *Tetramorium*  *Messor*  Dolichoderinae  *Plagiolepis*  Formicidae unident.  *Aphaenogaster*  *Messor*  Formicidae unident. | \| 5767 \| \| --- \| \| 5811 \| \| 786 \| \| 298 \| \| 71 \| \| 55 \| \| 4 \| \| 4 \| \| 2 \| \| 2 \| \| 2 \| | ------- | ------ | 234 |
| J46 | 11019 | *Tetramorium*  *Plagiolepis*  *Messor* (*M. barbarus*)  Dolichoderinae  *Tetramorium*  *Messor*  *Aphaenogaster*  *Tetramorium*  *Tetramorium*  *Tetramorium* | \| 10270 \| \| --- \| \| 266 \| \| 75 \| \| 39 \| \| 39 \| \| 4 \| \| 3 \| \| 2 \| \| 2 \| \| 2 \| | Lepidoptera | 4 | 313 |
| J47 | 8464 | *Tetramorium*  *Plagiolepis*  *Tapinoma*  *Messor* (*M. barbarus*)  *Messor*  *Messor*  Formicidae unident.  Dolichoderinae  Formicidae unident. | \| 6724 \| \| --- \| \| 266 \| \| 216 \| \| 62 \| \| 11 \| \| 6 \| \| 6 \| \| 6 \| \| 2 \| | ------- | ------ | 1165 |
| J48 | 5287 | *Tetramorium*  Dolichoderinae  Formicidae unident.  *Plagiolepis*  *Tapinoma*  Formicidae unident. | \| 2314 \| \| --- \| \| 948 \| \| 8 \| \| 6 \| \| 4 \| \| 2 \| | Lepidoptera  Lepidoptera | 893  118 | 994 |
| J49 | 24491 | *Messor* (*M. barbarus*)  *Messor*  Dolichoderinae  *Tetramorium*  *Plagiolepis*  *Messor*  *Aphaenogaster* | \| 21573 \| \| --- \| \| 421 \| \| 408 \| \| 159 \| \| 8 \| \| 6 \| \| 4 \| | ------- | ------ | 1912 |
| J50 | 17490 | *Tetramorium*  *Plagiolepis*  *Messor*  *Messor* (*M. barbarus*)  Dolichoderinae  Formicidae unident.  *Tetramorium*  Formicidae unident. | \| 14419 \| \| --- \| \| 2339 \| \| 530 \| \| 133 \| \| 59 \| \| 4 \| \| 2 \| \| 2 \| | ------- | ------ | 2 |
| J51 | 13603 | *Tetramorium*  *Messor* (*M. barbarus*)  *Messor*  Formicidae unident.  Dolichoderinae  *Plagiolepis* | \| 11106 \| \| --- \| \| 1988 \| \| 21 \| \| 17 \| \| 2 \| \| 2 \| | Lepidoptera | 2 | 465 |
| J52 | 9174 | *Tetramorium*  *Plagiolepis*  *Messor* (*M. barbarus*)  Dolichoderinae  *Aphaenogaster* | \| 5199 \| \| --- \| \| 678 \| \| 223 \| \| 80 \| \| 2 \| | ------- | ------ | 2992 |
| J53 | 19733 | *Tetramorium*  *Plagiolepis*  Dolichoderinae  *Messor* (*M. barbarus*) | \| 18956 \| \| --- \| \| 239 \| \| 157 \| \| 17 \| | Lepidoptera | 5 | 359 |
| J54 | 36374 | *Messor* (*M. barbarus*)  *Tetramorium*  *Messor*  Dolichoderinae  *Plagiolepis*  *Messor* | \| 26428 \| \| --- \| \| 5047 \| \| 1847 \| \| 309 \| \| 185 \| \| 2 \| | Lepidoptera | 272 | 2284 |
| Total | 1,159,115 | 1,058,557 | | 45,521 | | 54,846 |

**Table S4:** The results of primers laboratory tests with different annealing temperatures set up, evaluated based on the presence / absence of PCR product detected on agarose gels (+ = good PCR amplification / strong band on the gel, · = weak band on the gel, - = no PCR product amplified; ZBJ = „universal“ invertebrate primers, Form = ant specific primers).

| **Primers/ annealing temperature** | **ZBJ** | | | | | **ZBJ + Blocking oligo** | | | | | **Form** |
| --- | --- | --- | --- | --- | --- | --- | --- | --- | --- | --- | --- |
| **Species** | **48 °C** | **49 °C** | **50 °C** | **52 °C** | **54 °C** | **48 °C** | **49 °C** | **50 °C** | **52 °C** | **54 °C** | **50 °C** |
| *Callilepis nocturna* | + | + | + | · | - | · | · | · | - | - | - |
| *Callilepis schuszteri* | + | + | · | - | - | - | - | - | - | - | - |
| *Nomisia exornata* | + | + | N/A | N/A | N/A | - | - | N/A | N/A | N/A | - |
| *Myrmica* sp. | + | + | + | + | + | + | + | · | - | - | + |
| *Lasius flavus* | · | · | · | · | - | · | · | - | - | - | + |
| *Temnothorax* | · | · | · | - | - | · | · | · | - | - | + |
| *Tetramorium cf. caespitum* | · | · | - | - | - | · | · | - | - | - | + |
| *Camponotus vagus* | + | + | + | + | + | + | + | + | + | · | + |
| *Lasius platythorax* | · | · | · | · | - | · | · | · | - | - | + |
| *Camponotus aethiops* | + | + | N/A | N/A | N/A | + | + | N/A | N/A | N/A | + |
| *Messor barbarus* | + | + | N/A | N/A | N/A | + | + | N/A | N/A | N/A | + |
| *Cataglyphis hispanica* | + | + | N/A | N/A | N/A | + | + | N/A | N/A | N/A | + |
| *Tetramorium semilaeve* | - | - | N/A | N/A | N/A | - | - | N/A | N/A | N/A | + |
| *Aphaenogaster senilis* | + | + | N/A | N/A | N/A | + | + | N/A | N/A | N/A | + |
| *Acheta domestica* | - | - | N/A | N/A | N/A | - | - | N/A | N/A | N/A | - |
| *Ephestia kuehniella* | + | + | N/A | N/A | N/A | + | + | N/A | N/A | N/A | - |
| *Symploce pallens* | - | - | N/A | N/A | N/A | - | - | N/A | N/A | N/A | - |
| *Drosophila melanogaster* | + | + | N/A | N/A | N/A | + | + | N/A | N/A | N/A | - |
| *Callosobruchus maculatus* | + | + | N/A | N/A | N/A | + | + | N/A | N/A | N/A | - |
| *Reticulitermes* sp. | + | + | N/A | N/A | N/A | + | + | N/A | N/A | N/A | - |
| *Sinella curviseta* | + | + | N/A | N/A | N/A | + | + | N/A | N/A | N/A | - |
| *Clubiona* sp. | + | + | N/A | N/A | N/A | + | + | N/A | N/A | N/A | - |
| *Armadillidium vulgare* | · | · | N/A | N/A | N/A | · | · | N/A | N/A | N/A | - |
